# Changes in Obesity Odds Ratio among Iranian Adults, since 2000: Quadratic Inference Functions Method

**DOI:** 10.1155/2016/7101343

**Published:** 2016-10-10

**Authors:** Enayatollah Bakhshi, Koorosh Etemad, Behjat Seifi, Kazem Mohammad, Akbar Biglarian, Jalil Koohpayehzadeh

**Affiliations:** ^1^Department of Biostatistics, University of Social Welfare and Rehabilitation Sciences, Tehran, Iran; ^2^Center for Diseases Control, Ministry of Health and Medical Education, Tehran, Iran; ^3^Department of Physiology, Medicine School, Tehran University of Medical Sciences, Tehran, Iran; ^4^Department of Biostatistics, School of Public Health and Institute of Public Health Research, Tehran University of Medical Sciences, Tehran, Iran

## Abstract

*Background.* Monitoring changes in obesity prevalence by risk factors is relevant to public health programs that focus on reducing or preventing obesity. The purpose of this paper was to study trends in obesity odds ratios (ORs) for individuals aged 20 years and older in Iran by using a new statistical methodology.* Methods.* Data collected by the National Surveys in Iran, from 2000 through 2011. Since responses of the member of each cluster are correlated, the quadratic inference functions (QIF) method was used to model the relationship between the odds of obesity and risk factors.* Results.* During the study period, the prevalence rate of obesity increased from 12% to 22%. By using QIF method and a model selection criterion for performing stepwise regression analysis, we found that while obesity prevalence generally increased in both sexes, all ages, all employment, residence, and smoking levels, it seems to have changes in obesity ORs since 2000.* Conclusions.* Because obesity is one of the main risk factors for many diseases, awareness of the differences by factors allows development of targets for prevention and early intervention.

## 1. Background

Obesity has become a growing serious health problem in all regions of the world and is linked to increased risk of cardiovascular diseases [1–4], diabetes [5], chronic pain [6], arthritis [7], asthma [8], Alzheimer's disease [9], and functional impairment [10] and so it can affect a person's quality of life. Since 1980, obesity has more than doubled in the world and studies showed the association with higher mortality [11]. In 2014, more than 600 million of world's population and, specifically, 13% of adults aged 18 and over were obese [12]. In 2014, more than 36% of US adults were obese. The prevalence of obesity increased among US adults and youth from 1998 through 2014. However, there was no significant change from 2003-2004 to 2013-2014 in obesity among the youth, and the prevalence of obesity remains high [13]. In Canada, the prevalence of obesity among adults increased 200% (from 6.1% to 18.3%) between 1985 and 2011 [14]. Results from the Australian Health Survey (2011-12) showed that almost 28% of the population aged 18 and over were obese. In England, the obesity prevalence among adults rose from 14.9% to 25.6% from 1993 through 2014 [15].

The relationship between obesity and various factors was explored. Potential predictors could be age and sex [16]. Another potential confounder of this association in adults includes the socioeconomic status [17]. Significant association between obesity and place of residence was observed in studies [18, 19]. The association of obesity with lifestyle habits such as smoking has been analyzed in some studies [20].

Monitoring changes in obesity prevalence by risk factors is relevant for public health programs that focus on reducing or preventing obesity. There is no study to explore changes in obesity ORs for related factors in Iranian adults. The purpose of this paper was to study trends in obesity ORs for individuals aged 20 years and older in Iran.

## 2. Methods

### 2.1. Data

#### 2.1.1. NHSI 2000

The National Health Survey in Iran (NHSI) was designed by the Iranian Ministry of Health and Medical Education to determine related factors with public health. Targeted population was all Iranian citizens. Household was defined as those living in the same residence, participating in the households expenses and usually eating together. Any individual living single was also considered a household. Cluster method was the sampling way, 8 households in each. This number for the cluster size was based on one-day performance capacity of the data collection group: four persons (2 physicians, 1 interviewer, and 1 lab technician). The information about households was achieved from the Family Questionnaire during an in-home interview [21, 22]. The NHSI data used in this analysis included 27869 participants aged +20 years.

#### 2.1.2. WHO Step Surveys 2007–2011

The WHO STEPwise approach to Surveillance (STEPS) is a simple, standardized method for collecting, analyzing, and disseminating data in WHO member countries. To compare between WHO member countries, the same standardized questions were used [23].

This survey in Iran was also conducted under the supervision of WHO and approved by the ethics committee of the Center for Disease Management located in the Iranian Ministry of Health and Medical Education. Representative data for adults aged ≥15 was achieved by cluster sampling method and in collaboration with 31 Iranian medical schools. The number of clusters in each province was proportional to the size of that province, with each cluster including 10 men and 10 women. Step surveys data included 26716, 20917, and 8425 participants aged +20 years from 2007, 2009, and 2011, respectively.

Pregnant women were excluded from the analyses.

## 3. Measurements

### 3.1. Response Variable

For all four surveys, height and weight were actually measured rather than self-reported. Height was measured without shoes to the nearest 5 mm. Weight was measured to the nearest 0.1 kg with the subject in light indoor clothes, with emptied pockets and without shoes. BMI (body mass index) was calculated as weight in kilograms divided by height in meters squared, and obesity was defined as BMI of 30.0 or higher.

### 3.2. Independent Variables

For state comparisons between all four studies, we limited independent variables to age, sex, place of residence, employment, and smoking status.

Information about the respondents' age was based on their self-reported birth year, and subjects were stratified into five 10-year age groups (20–29, 30–39, 40–49, 50–59, and +60 years). The subjects were grouped according to their place of residence as living in cities (urban) or villages (rural). Employment status was analyzed as three categorical variables (public employed/private employed/other (student, retired, home maker, unable to work, soldier, and others)). Smoking status was dichotomized into smoker versus nonsmoker.

### 3.3. Statistical Analysis

Logistic regression has become a popular approach for analyzing binary data. It is a common characteristic of such method that observations can be assumed to be statistically independent. Since responses in the same cluster are typically more similar than responses from different clusters, the analytic approach for modeling this type of data is generalized estimating equations (GEE) method, which takes intracluster correlation into account rather than assuming independency. Selection of a working correlation structure is at the discretion of the researcher. However, a misspecified working correlation structure affects efficiency [24].

The quadratic inference functions (QIF) method proposed by Qu et al. [25] is alternative to the GEE approach, which enables us to do goodness-of-fit tests and model selection [26]. We applied QIF method to assess the association between obesity and all other factors. All analyses were carried out by using the SAS software package. The results are presented as the odds ratios and their 95% confidence intervals (CIs).

### 3.4. QIF Method

A common assumption for GEE is that the outcomes from different clusters are independent and within clusters are dependent. Let *Y*_*ij*_ be the binary response and let *X*_*ij*_ be a vector of covariates from the* j*th individual in the *i*th cluster, for *K* clusters. The GEE model has two parts: (i) the marginal mean *μ*_*ij*_ is considered as a function of the covariates through a link function *g* with *g*(*μ*_*ij*_) = *g*(*E*(*Y*_*ij*_∣*X*_*ij*_)) = *X*_*ij*_′*β*; and (ii) the variance of *μ*_*ij*_ is a function of the mean Var(*Y*_*ij*_∣*X*_*ij*_) = *φV*(*μ*_*ij*_), where *φ* is a scalar parameter. The basis of quasi-likelihood estimation is that the entire distribution of the responses is not needed, and the equations are completely specified by the mean and variance of the random response. The GEE estimator *β* is the solution of these equations:

(1) $$\begin{array}{c} {\sum_{i}\left(\frac{\partial \mu_{i}}{\partial \beta }\right)}^{\prime }{\left(\;D_{i}^{1/2}R_{i}\left(\;\alpha \;\right)D_{i}^{1/2}\;\right)}^{-1}\left(\;y_{i}-\mu_{i}\;\right)=0. \end{array}$$

*D*_*i*_ is the diagonal matrix of the marginal variances, and *R*_*i*_(*α*) is the working correlation matrix.

In QIF method, we consider the inverse of working correlation structure as a linear combination of several basic matrices:

(2) $$\begin{array}{c} R^{-1}\approx a_{0}I+a_{1}M_{1}+\cdots +a_{k}M_{k}, \end{array}$$

where *M*_0_ is the identity matrix and *M*_*i*_ are known basis matrices. While determining that a working correlation structure is difficult, we can also select a hybrid working correlation by combining basic matrices from several working correlations [27].

Substitute (2) into (1) and rearrange slightly to obtain

(3) g−Kβ=1K∑iKgiβ≈1K∑i∂μi∂β′Di−1yi−μi∑i∂μi∂β′Di−1/2M1Di−1/2yi−μi⋮∑i∂μi∂β′Di−1/2MmDi−1/2yi−μi.

That is, (1) takes the form of $K{\left(a_{0},a_{1},\ldots ,a_{m}\right)}^{\prime }{\overset{-}{g}}_{K}(\beta )$. It is a problem to solve it for *β*, because the number of equations is greater than the number of parameters. By using generalized method of moments [27], we can minimize the QIF:

(4) QIFKβ=Kg−KβK−1∑iKgiβgi′β−1g−Kβ.

Note that the QIF_K_(*β*) contain only the regression parameter *β* and the basis matrices *M*_*i*_.

## 4. Results

We estimated 2000 to 2011 trends in the prevalence of obesity by sex, age, place of residence, employment, and smoking status for Iranian population.

Table 1 provides the estimated obesity prevalence rates for the Iranian adults from the four data sources. Not surprisingly, the prevalence of obesity nearly doubled from 12% in 2000 to 20% in 2007 and rose slightly to 22% between 2009 and 2011, and the prevalence of obesity remains high. From 2000 to 2011, increases in obesity among Iranian adults continue in both sexes, all ages, all employment levels, both places of residence levels, and both smoking levels.

Table 2 shows the OR (odds ratio) of the QIF regression method for 2000, 2007, 2009, and 2011 data. In all four surveys, obesity was significantly associated with age, sex, place of residence, employment levels, and smoking status.

Using age group 20–30 years as the reference, obesity ORs for 30–40 were 2.44 (95% CI 2.18 to 2.72), 2.61 (95% CI 3.35 to 2.91), 2.42 (95% CI 2.14 to 2.72), and 2.16 (95% CI 2.81 to 2.59) in 2003, 2007, 2009, and 2011, respectively; obesity ORs for 40–50 years were 3.52 (95% CI 3.13 to 3.94), 3.66 (95% CI 3.30 to 4.06), 3.74 (95% CI 3.23 to 4.20), and 3.41 (95% CI 2.86 to 4.08) in 2003, 2007, 2009, and 2011, respectively; obesity ORs for 50–60 were 3.40 (95% CI 2.98 to 3.88), 3.52 (95% CI 3.17 to 3.90), 3.52 (95% CI 13 to 3.94), and 3.25 (95% CI 2.74 to 3.86) in 2003, 2007, 2009, and 2011, respectively; obesity ORs for 60+ years were 3.02 (95% CI 1.78 to 2.31), 3.25 (95% CI 2.86 to 3.69), 3.15 (95% CI 2.72 to 3.65), and 2.82 (95% CI 2.29 to 3.46) in 2003, 2007, 2009, and 2011, respectively.

Compared with male, obesity ORs for female were 2.40 (95% CI 2.10 to 2.74), 2.35 (95% CI 2.15 to 2.58), 2.57 (95% CI 2.36 to 2.79), and 1.58 (95% CI 1.34 to 1.85) in 2003, 2007, 2009, and 2011, respectively. Obesity ORs for urban adults were 1.98 (95% CI 1.81 to 2.16), 1.63 (95% CI 1.53 to 1.74), 1.49 (95% CI 1.39 to 1.61), and 1.46 (95% CI 1.29 to 1.65) in 2003, 2007, 2009, and 2011, respectively. Obesity ORs for public employed people were 0.72 (95% CI 0.61 to 0.84), 0.76 (95% CI 0.67 to 0.87), 0.82 (95% CI 0.70 to 0.95), and 0.55 (95% CI 0.45 to 0.67) in 2003, 2007, 2009, and 2011 respectively; obesity ORs for private employed people were 0.84 (95% CI 0.73 to 0.97), 0.93 (95% CI 0.84 to 1.02), 0.74 (95% CI 0.58 to 0.965), and 0.77 (95% CI 0.64 to 0.92) in 2003, 2007, 2009, and 2011, respectively, compared with others. Compared with nonsmokers, obesity ORs for smokers were 0.65 (95% CI 0.56 to 0.76), 0.69 (95% CI 0.63 to 0.77), 0.77 (95% CI 0.68 to 0.88), and 0.57 (95% CI 0.46 to 0.69) in 2003, 2007, 2009, and 2011, respectively.

## 5. Discussion

**Figure 1:**
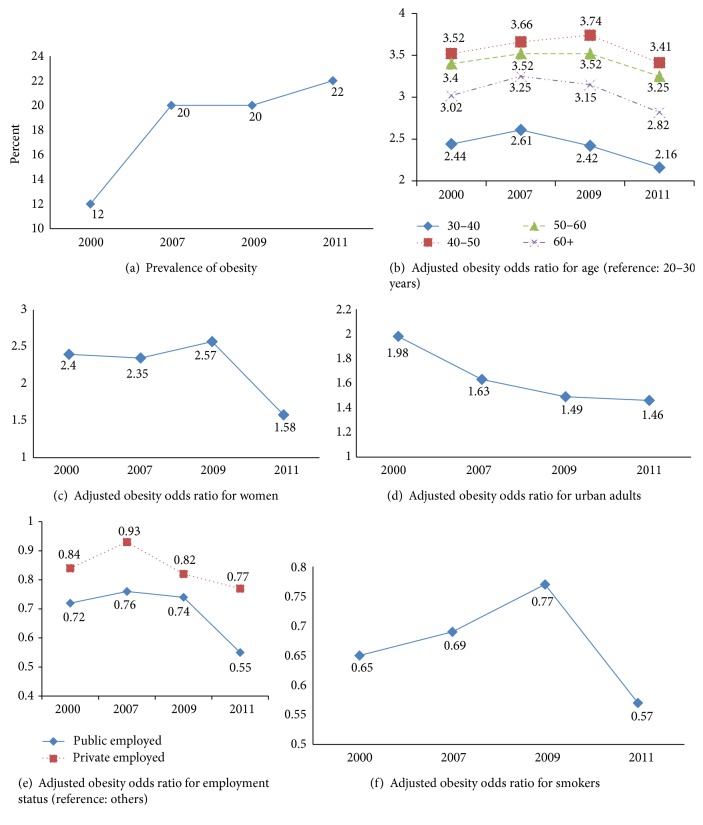
Prevalence of obesity (a) and association with some factors (b–f) by using QIF method, national surveys, 2000–2011.

In almost ten-year period from 2000 to 2011, the prevalence of obesity among Iranian adults doubled. Precisely, trends in obesity prevalence show alarming increase among adults from 2000 through 2007 but slow increase from 2007 through 2011. From 2000 to 2007, the prevalence of obesity in adults has jumped markedly in all age groups, both men and women, all employment levels, both places of residence levels, and both smoking levels (Figure 1(a)).

Similar to some studies [28–33], our results showed positive association between age and obesity. With progression of age, there are changes in food intake, energy expenditure, and appetite and body composition in addition to bone and muscle losses that influence the body composition. Although fat-free mass (FFM) progressively decreases after the age of 30, fat mass increases. FFM decreases by up to 40% from the age of 20 to 70 primarily to skeletal muscle. However, FFM reaches its peak at the age of 20–30 years; the maximal fat mass is usually gained at the age of 60–70. Both kinds of fats subsequently decline after this age. Overall, our findings also show a continuing decrease in the age differences in the obesity OR after 2009 (Figure 1(b)).

The results from our analysis show that the prevalence of obesity is higher in females and the difference by sex is steadily decreasing (Figure 1(c)). Pregnancy and menopause are the significant factors in the development of obesity for many women. Some studies showed that the average woman gains 1–2.5 kg during menopausal transition [34] and more than 10 kg after delivery [35]. Flegal et al. [36] showed significant increasing linear trends among women for overall obesity but not among men in the United Sates, between 2005 and 2014. The one factor most consistently related to weight gain is physical activity. Changes in family work patterns may be concerned. Both men and women are busy and have less time to spend on health behaviors and reduced time for cooking, and meals eaten away from home have contributed to diets becoming increasingly high in fat and energy.

Our findings also show a continuing decrease in the regional differences in the prevalence of obesity (Figure 1(d)), although the prevalence of obesity was higher in urban areas, results similar to findings reported in some studies that have shown that those who lived in rural areas had a lower risk of becoming obese [37]. Differences in the prevalence of obesity across places of residence may be dependent on whether developed or developing countries are studied [18]. Some studies show that, in developing countries, urban residents are taller, are heavier, and have a higher BMI than those who live in rural areas [38]. In rural areas of the developing world, people make major contributions to agricultural production, so the opportunities for outdoor activities and an active lifestyle are likely to be greater in urban environments.

The prevalence of obesity rose according to level of employment. The prevalence in public employed level was lower than the other levels. Our findings also show that the difference by employment level is steadily increasing (Figure 1(e)). This difference may be due to physical activity. It is also possible that there is more discrimination against the obese or obese people may end up in lower status jobs through stronger selective processes in Iran. Obesity may be more acceptable among unemployed people. It is commonly believed that overweight and obese people are lazy and gluttonous and they lack self-control. Some people believe that an obese person is taking up more space than he or she should and a job is often denied because of their weights [39]. Another explanation for the effect of the workforce may involve energy expenditure at work or the structured lifestyle that active people impose.

Our results are consistent with the founding of some studies that smoking is related to decreased obesity odds among adults [40]. From 2000 to 2009, this difference was decreasing, whereas this difference continues to rise after 2009 (Figure 1(f)). Biological and psychological factors could be related to the effect of smoking on obesity. Studies found that nicotine-induced decreases in appetite are due to hypothalamic melanocortin system [41, 42]. Another study showed that some tobacco companies had added some substances into their cigarette in order to reduce smokers' appetite [43]. An increase of energy expenditure while smoking, both in resting and in light physical activity conditions, may relate to lower prevalence of obesity in smokers [24]. Loos [44] entered a new era of gene discovery for obesity.

The use of cross-sectional surveys is a limitation of this study. So we were unable to draw conclusions regarding the causal association between factors and obesity. Longitudinal data would provide a more valid and reliable estimate of the prevalence of obesity and related factors. A further limitation is that physical activity and marital status were not used in our investigation.

The use of large, nationally representative data sets is a major strength of our study. Because people tend to underestimate their weight and overestimate their height [45], height and weight were actually measured rather than self-reported.

## 6. Conclusions

While obesity prevalence generally increased in both sexes, all ages, all employment, residence, and smoking levels, it seems to have changes in obesity ORs since 2000. Because obesity is one of the main risk factors for many diseases, awareness of the differences by factors allows development of targets for prevention and early intervention.

## Figures and Tables

**Table 1:** Descriptive prevalence of obesity across study variable levels in the four studies.

Variable	2000			2007			2009			2011		
	Rate^a^	%	Total	No	%	Total	No	%	Total	No	%	Total
*Age*												
20–30	556	6.4	8674	600	9.7	6160	493	9.4	5226	263	10.5	2500
30–40	969	13.6	7115	1214	20.5	5922	908	19.4	4671	354	21.2	1666
40–50	906	18.3	4948	1578	26.1	6047	1222	26.6	4599	423	31.1	1361
50–60	496	18.0	2753	1551	25.6	6050	1201	25.7	4676	592	29.4	2017
60+	491	11.2	4379	603	23.8	2537	409	23.4	1745	246	27.9	881
*Sex*												
Men	832	6.6	12690	1698	12.9	13194	1235	12.2	10083	520	14.6	3551
Women	2586	17	15179	3848	28.5	13522	1998	27.7	10834	1358	27.9	4874
*Place of residence*												
Rural	785	8.0	9833	1807	16.5	10920	1503	16.9	8909	457	18.4	2478
Urban	2633	14.6	18036	3739	23.7	15796	2730	22.7	12008	1421	23.9	5947
*Employment status*												
Other	2574	16.0	16123	4117	25.1	16370	3916	20.9	18705	1370	29.5	4648
Public employed	239	8.7	2741	369	15.8	2331	246	15.6	1574	166	11.4	1457
Private employed	605	6.7	9005	1060	13.2	8015	71	11.1	638	342	14.7	2320
*Smoking status*												
Nonsmoker	3175	13.3	23821	4819	22.9	21076	3827	21.7	17629	1728	23.9	7228
Current smoker	243	6.0	4048	727	12.9	5640	406	12.3	3288	150	12.5	1197

*Total*	3418	12	27869	5546	20	26716	4233	20	20917	1878	22	8425

^a^Prevalence of obesity.

**Table 2:** Adjusted^a^ obesity odds ratios in four surveys of Iranian adults.

Variable	2000		2007		2009		2011	
	OR^b^	95% CI^c^	OR^b^	95% CI^c^	OR^b^	95% CI^c^	OR^b^	95% CI^c^
*Age*								
20–30	1.00		1.00		1.00		1.00	
30–40	2.44	2.18–2.72	2.61	2.35–2.91	2.42	2.14–2.72	2.16	1.81–2.59
40–50	3.52	3.13–3.94	3.66	3.30–4.06	3.74	3.23–4.20	3.41	2.86–4.08
50–60	3.40	2.98–3.88	3.52	3.17–3.90	3.52	3.13–3.94	3.25	2.74–3.86
60+	3.02	1.78–2.31	3.25	2.86–3.69	3.15	2.72–3.65	2.82	2.29–3.46
*Sex*								
Men	1.00		1.00		1.00		1.00	
Women	2.40	2.10–2.74	2.35	2.15–2.58	2.57	2.36–2.79	1.58	1.34–1.85
*Place of residence*								
Rural	1.00		1.00		1.00		1.00	
Urban	1.98	1.81–2.16	1.63	1.53–1.74	1.49	1.39–1.61	1.46	1.29–1.65
*Employment status*								
Other	1.00		1.00		1.00		1.00	
Public employed	0.72	0.61–0.84	0.76	0.67–0.87	0.74	0.58–0.96	0.55	0.45–0.67
Private employed	0.84	0.73–0.97	0.93	0.84–1.02	0.82	0.70–0.95	0.77	0.64–0.92
*Smoking status*								
Nonsmoker	1.00		1.00		1.00		1.00	
Current smoker	0.65	0.56–0.76	0.69	0.63–0.77	0.77	0.68–0.88	0.57	0.46–0.69

^a^Adjusted for all other variables in the table.

^b^Odds ratio.

^c^Confidence interval.

## Abbreviations

BMI: Body mass index
OR: Odds ratio
NHSI: National Health Survey in Iran
WDICH: Without dichotomizing
DICH: Dichotomizing.
